# Comparative biochemical properties of recombinant goat and calf chymosins and their implications in dairy processing

**DOI:** 10.1038/s41598-025-11833-x

**Published:** 2025-07-21

**Authors:** Zhiger Akishev, Madina Auyez, Annelya Tursunbekova, Bekbolat Khassenov

**Affiliations:** 1 National Center for Biotechnology, 13/5 Korgalzhyn Road, Astana, 010000 Kazakhstan; 2 S. Seifullin Kazakh Agro Technical Research University, 62 Zhenis Avenue, Astana, 010001 Kazakhstan; 3 “GenLab” LLP, 19/1, 69, M. Gabdullin Street, Astana, 010000 Kazakhstan

**Keywords:** Chymosin, *Pichia pastoris*, Recombinant DNA, Milk-clotting activity, Goat milk, Biochemistry, Biotechnology, Molecular biology

## Abstract

The nutritional and functional properties of goat milk are well-established. However, the use of camel and calf-derived chymosins during cheese making from goat milk results in relatively low productivity. This study aimed to produce recombinant goat chymosin and investigate its biochemical properties and milk-clotting activity in goat and cow milk. To achieve this, recombinant goat chymosin was successfully expressed in *Pichia pastoris*, yielding 148,000 units/L of enzyme activity. Recombinant goat chymosin has exhibited maximum enzyme activity at 60 °C in both milk types, whereas recombinant calf chymosin showed maximum activity at 65 °C for goat milk and 75 °C for cow milk. Moreover, goat chymosin exhibited 13.6% higher activity in goat milk than that in cow milk. Both the enzymes efficiently clot sheep, cow, and camel milk but exhibited low activity against mare milk. The milk-clotting activity of both the enzymes was remarkably influenced by the calcium chloride concentration. To our knowledge, this study is the first to compare the biochemical properties of recombinant goat and calf chymosin in goat milk. The high specific activity of recombinant goat chymosin in goat milk suggests its potential as an effective milk-clotting enzyme in goat milk cheese production.

**Supplementary Information:**

The online version contains supplementary material available at 10.1038/s41598-025-11833-x.

## Introduction

Chymosin (EC 3.4.23.4) is a pepsin-like aspartic protease used in cheese production^1^. It is a highly specific endopeptidase hydrolyzing the bond between Phe^105^ and Met^106^ in the κ-casein molecule^2^. This reaction destabilizes the casein micelles in milk, resulting in a loss of solubility and precipitation of casein proteins. This characteristic of chymosin makes it a key coagulating enzyme in the cheese-making process. Chymosin also exhibits proteolytic activity which is significantly lower than that of pepsin^3^. These dual activities allow the use of chymosin as a key coagulation enzyme in cheese making^1^.

Chymosin is formed over 10 days in newborn mammals and is secreted in an inactive form, prochymosin^3^. Chymosin activation occurs at low pH (3.0–3.5) and is accompanied by the removal of a 42 amino acid propeptide, resulting in active chymosin. This activation process can be replicated outside the stomach, allowing large-scale production of chymosin via recombinant DNA technology in microorganisms^1,4^. In addition to the popular calf chymosin, recombinant chymosins from marals, elks, yaks, and camels are produced by microorganisms^5–10^. Chymosins can also be produced in bacteria, fungi, and yeast^4,6,8,9,11–13^.

Bovine milk is the predominant source for industrial cheese production. The total proportion of cow and buffalo milk production remained almost constant at 96%, whereas goat milk production in 2020 was 2.3% of the global total (https://www.fao.org/faostat/en/#search/milk). Goat milk plays an important role in agriculture in the Mediterranean, Foreland, and Southeast Asian regions^14^, where the availability of cow milk is limited^15^. The world goat population has already surpassed one billion, of which an estimated 203 million are classified as dairy goats, producing 15.26 million tons of milk annually. Asia accounts for more than 50% of the world’s goat milk supply, mainly contributed by the populous countries of India, Pakistan, and Bangladesh^16^. The remarkable nutritional and functional characteristics of goat milk, its buffering properties, and moderate alkalinity relative to cow milk^17^ have highlighted the potential of developing goat milk-based novel products^18^. The ratio of casein and whey proteins differs between goat and cow milk, which also accounts for differences in functional properties^17^. Camel and calf chymosins exhibit lower milk-clotting activity in goat milk than that in cow milk^9^. Alignment of the amino acid sequences of related chymosins revealed that goat chymosin differs from calf chymosin at 18 positions. Presumably, goat chymosin has a higher milk-clotting activity in goat milk, which will favor its use in the production of cheese and curd from this type of milk. Therefore, this study aimed to produce recombinant goat chymosin and investigate its biochemical properties and milk-clotting activity relative to calf chymosin in goat and cow milk.

## Materials and methods

### Strains, enzymes, oligonucleotides, vectors, mediums, and chemicals

To utilize the strong, highly-inducible P_AOX1_ promoter for expression of protein the pPICZαA vector was used to construct the transforming cassette and for secreted expression in *Pichia pastoris* GS115 strain (Invitrogen, Carlsbad, CA, USA). Phusion High-Fidelity DNA polymerase was chosen for the error-prone amplification, *Eco*RI, *Not*I endonucleases were chosen due to the presence of unique restriction sites flanking the cloning site in the pPICZαA vector and their compatibility with our cloning strategy, ensuring directional insertion of the gene. FastAP phosphatase and T4 DNA ligase enzymes were selected for cloning the chymosin gene, *Mss*I endonuclease for linearization of yeast transforming cassette, and *Escherichia coli* DH5α strain was selected for DNA manipulations (Thermo Fisher Scientific, Vilnius, Lithuania).

The chemical reagents used in this study were of molecular biological or analytical grade and were were obtained from commercial suppliers (Sigma-Aldrich, St. Louis, MO, USA; AppliChem, Darmstadt, Germany; Cytiva, Uppsala, Sweden) unless otherwise specified. Previously purified recombinant calf chymosin (rBtCYM)^9^ was used in this study. Skimmed cow and goat milk was purchased from Bellakt shop (Minsk, Belarus). Fresh, unpasteurized milk from cow, goat, ewe, camel and mare were purchased at local city markets.

### Yeast vector with goat prochymosin gene construction

The nucleotide sequence of the goat prochymosin (*Capra hircus*) gene (*procymCH*) was obtained from GenBank (accession number NM_001285759.1). The complete gene sequence was synthesized in native form by Macrogen (Seoul, Korea). The *procymCH* gene was amplified with oligonucleotides KidCYMfw (5′-CCGGAATTCGCTGAGATCACCAGGATCC-3′) and KidCYMrv (5′-ATAGTTTAGCGGGGCCGCGCGATGGCTTTTTGGCCAGCCCCCCA-3′) and cloned in the pPICZαA vector at the *Eco*RI and *Not*I sites, within the frame α-factor secretion signal for extracellular protein production, to obtain the shuttle plasmid pPICZαA/ProcymCH.

### Yeast strain transformation

To obtain a transforming cassette, the pPICZaA/ProcymCH vector was linearized with *Mss*I endonuclease at the AOX1 locus for efficient integration into the *Pichia* genome. The linearized vector was purified using phenol/chloroform extraction, followed by ethanol precipitation. Freshly prepared competent *P. pastoris* GS115 cells were transformed using the EasySelect^™^ Yeast *Pichia* Expression Kit. Three micrograms of a transforming cassette with the *procymCH* gene was added to 80 µL of cell suspension and pulsed in a 0.2 cm electroporation cuvette with the pulse of 2 kV (10 kV cm^−1^) for 4.8 ms pulse time using a Bio-Rad MicroPulser^™^ electroporator (Hercules, CA, USA). *P. pastoris* GS115/pPICZαA/ProcymCH transformant clones were selected on Petri dishes with YEP (1% yeast extract, 2% peptone, pH 7.0), 3% glucose, 2% agar, and zeocin (200 µg/mL) for selection. The selected clones were tested for the presence of PCR insertion with primers AOX1fw (5′-GACTGGTTCCAATTGACAAGC-3′) and AOX1rv (5′-GCAAATGGCATTCTGACATCC-3′) with the expected product size of 1640 bp. The insertion-positive clones were cultured in YEP broth with 1% methanol for 3 d and analyzed for milk-clotting activity. The clone with maximum activity was selected as the recombinant chymosin-producing strain.

### Prochymosin flask culture expression

The *P. pastoris* GS115/pPICZαA/ProcymCH strain cells were inoculated into a 50 mL flask containing 5 mL of YEPD medium (1% of yeast extract, 2% of peptone, and 3% of dextrose, pH 7.0) with zeocin (100 µg/mL) and were cultured for 16 h at 30 °C and 250 rpm in the shaking incubator. The overnight culture was inoculated into 50 mL of YEPD in a 500 mL shake flask and incubated at 30 °C and 250 rpm overnight. The overnight culture was inoculated into 500 mL of YEPD and supplemented with 100 mM citrate-phosphate buffer pH 4.0, 10 mM ascorbic acid, 1.34% YNB, 0.0004% Biotin, and 5% sorbitol to OD_600_ of 2 and grown using the same culture conditions in a shaking incubator Climo-Shaker ISF1-X (Kuhner, Basel, Switzerland) at 28 °C and 250 rpm for 120 h with daily addition of 2% methanol. The cells were collected using centrifugation at 5000 × g, for 20 min, at 4 °C, and discarded; the supernatant was used for enzyme purification.

### Purification of Recombinant goat chymosin

The yeast culture supernatant was centrifuged (10000 × g, 30 min, 4 °C), filtered (0.22 μm) and the clarified supernatant was used to purify recombinant goat chymosin (rChCYM). The pH of the supernatant was lowered to 3.0, using 1 M HCl, and loaded onto an SP-Sepharose FF (strong cation exchanger) column pre-equilibrated with 25 mM NaCl in 50 mM sodium citrate buffer (pH 3.0). The column was washed with 50 mM NaCl in 25 mM sodium acetate buffer (pH 5.5). The bound fraction was eluted with 750 mM NaCl in 25 mM sodium acetate buffer (pH 5.5). A second purification step involving anion-exchange chromatography on Q-Sepharose was used to further enhancing purity and produce a better-quality, more concentrated enzyme. The NaCl concentration in the eluted fraction was reduced by dilution to 25 mM and the mixture was loaded onto a Q-Sepharose FF column previously equilibrated with 25 mM NaCl in 25 mM sodium acetate buffer (pH 5.5). The column was washed with the same buffer, and rChCYM was eluted with 10 column volumes of 50 mM to 2 M NaCl in 25 mM sodium acetate buffer (pH 5.5) gradient. Milk-clotting activity of the fractions was examined to select the most active fractions. All collected fractions were analyzed for presence of ~ 40–42 kDa protein using Sodium dodecyl sulphate-polyacrylamide gel electrophoresis (SDS-PAGE). The fractions with the highest milk-clotting activity were combined and used for further experiments.

### Milk-clotting assay

This assay was performed according to a previous study^8^ using 12% (w/v) reconstituted skimmed cow or goat milk dissolved in 0.1 mM sodium acetate buffer (pH 5.5) as a substrate. The reaction was performed in tubes containing 1 mL of substrate and 20 µL of enzyme solution at 37 °C. The time required for milk clot formation was also recorded. The amount of enzyme required to clot 1 mL of skimmed milk in 40 min (2400 s) at 37 °C was counted as a unit of milk clotting activity. For this purpose, chymosin activity units (A) was calculated using proportion with the following Eq. (1):

1 $$\:\text{A}=\frac{{V}_{milk}}{{V}_{chymosin}}\times\:\frac{2400s}{{T}_{mc}}$$

Where V_*milk*_ is the volume of milk (mL), V_*chymosin*_ is the volume of added chymosin (mL), and T_*mc*_ is the milk lotting time (s).

The milk-clotting activity on different milk types was measured by using freeze-dried fresh skimmed milk. Fresh milk was preheated to 40 °C, defatted by centrifugation at 3500 × g for 15 min (Eppendorf Centrifuge 5415R, Hamburg, Germany) and freeze-dried in BETA 2–8 LDplus (Christ, Osterode, Germany) at − 90 °C in vacuum (0.030 mBar) for 48 h. The dry milk was powdered.

Powdered milk was reconstituted at final composition of the substrate solution was brought to next: 10% (for mare’s) or 12% (for cow’s, ewe’s, goat’s and camel’s) milk, 0.1 M sodium acetate buffer (pH 5.5), 30 mM CaCl2. Clotting time in each tube was measured by time first flakes observed by inversion of tube. The experimental tests were conducted in triplicate, and the mean value was determined as the average of the three repetitions.

### Impact of substrate pH on milk clotting activity

The milk clotting activity was measured in the pH range of 4.5-8.0. Skimmed milk powder was dissolved either in 100 mM sodium acetate buffer (pH 4.5–6.0) or in 100 mM imidazole HCl buffer (pH 6.0–8.0). The condition that yielded the highest activity within that specific experimental series was set as 100%, and other activities were calculated relative to this maximum. The experimental tests were conducted in triplicate, and the mean value was determined as the average of the three repetitions.

### Impact of substrate temperature on milk clotting activity

The milk clotting activity was measured in the temperature range of 0–75 °C (with intervals of 5 °C) skimmed milk powder dissolved in 100 mM sodium acetate buffer (pH 5.5). The substrate and enzyme solutions were preheated to the reaction temperature for 3 min. The condition that yielded the highest activity within that specific experimental series was set as 100%, and other activities were calculated relative to this maximum. The experimental tests were conducted in triplicate, and the mean value was determined as the average of the three repetitions.

### Effect of CaCl_2_ on milk-clotting activity

The effect of additional calcium ions on milk-clotting activity was determined according to a previously described procedure^8^. The milk-clotting activity was studied at 37 °C measuring clotting time of milk substrate in the presence of CaCl_2_ in the concentration range from 0 to 160 mM dissolved in 100 mM sodium acetate buffer with pH 5.5. Enzyme activity in the absence of additional calcium ions was considered 100%, and the remaining samples with additional calcium ions were tested relative to this. The experimental tests were conducted in triplicate, and the mean value was determined as the average of the three repetitions.

### Effect of metal ions on the milk-clotting activity

The effect of ions of various metals on milk-clotting activity was studied at 37 °C in the presence of each of the following 12 metal ions: CaCl_2_, CoCl_2_, NiCl_2_, FeSO_4_, BaCl_2_, ZnCl_2_, MgCl_2_, MnCl_2_, CdCl_2_, LiCl. KCl, and NaCl (10 mM) were dissolved in 100 mM sodium acetate buffer (pH 5.5). The activity of the enzyme in the absence of metal ions was considered 100%, and the remaining samples were tested relative to this with various metal ions. The experimental tests were conducted in triplicate, and the mean value was determined as the average of the three repetitions.

### Proteolytic activity assay

Proteolytic activity was measured according to the method described by Anson^19^ with modifications. The reaction mixture consisted of 1 mL 1% hemoglobin in 50 mM citrate buffer (pH 3.0) and 0.02 mL enzyme. The mixture was incubated for 10 min at 37 °C. The reaction was stopped by adding 10% trichloroacetic acid (0.5 mL). The optical density was measured at 280 nm using a UV-1900i spectrophotometer (Shimadzu, Kyoto, Japan) the amount of released tyrosine was measured using standard curve. The amount of enzyme required to release 1 µg of tyrosine per minute was considered as unit activity. The experimental tests were conducted in triplicate, and the mean value was determined as the average of the three repetitions.

### Protein concentration determination

Protein concentration was determined using the Bradford method^20^ with bovine serum albumin as the standard. Here, 100 µL of Bradford reagent (protein analysis dye; Bio-Rad, Munich, Germany) and 860 µL of 10% phosphate buffered saline were mixed with 1% glycerol, and 40 µL of a protein sample were added. The mixture was incubated for 3 min at room temperature (20–23 °C), and the optical density was measured using a spectrophotometer at 595 nm. The measurements were carried out in three repetitions, with the average of the three repetitions being reported as the specified result.

### Recombinant goat chymosin production by fermentation in a bioreactor

A single colony was inoculated into 10 mL of YEPD broth and grown at 28 °C and 250 rpm for 24 h. Following this, the culture was sequentially grown under the same conditions for 24 h each as follows: transferred to 100 mL YEPD broth, transferred to 300 mL of YEPD medium, and then required number of cells inoculated into 5 L of YEPM medium to achieve a starting OD_600_ of 2 in a 10-L bioreactor (Biostat, Sartorius, Germany). The following cultivation conditions were used to operate the fermenter: 28 °C, 400 rpm, aeration 2–4 L/min, pH 4.0, with daily addition of 1% methanol. The culture period was 144 h. During fermentation, 50 mL aliquots were taken every 24 h, to measure milk-clotting activity and determine biomass growth.

### Alignment of the amino acid sequences of goat and bovine chymosins

Alignment of full-length amino acid sequences of *Capra hircus* (accession number NM_001285759.1) and *Bos taurus* (accession number j00003.1) chymosins was conducted using the Vector NTI Advance 11.0 (Invitrogen, Carlsbad, CA, USA) software.

### Preparation of cheese with Recombinant goat chymosin

Laboratory-scale cheese production was performed following as previously described^9^. Starter cultures and salts were excluded from the procedure because the purpose of this experiment was to test the effect of recombinant goat chymosin on the process of making cheese from goat and cow milk. Milk composition was determined using a Lactan 600 Ultra milk analyzer (Sibagropribor LLC, Novosibirsk, Russia) and normalized to 2% fat content. Milk was pasteurized at 75 °C for 30 s and cooled in ice to 35 °C. Following this, 5000 U of recombinant goat chymosin and 5 mM CaCl_2_ were added to 5 L of pasteurized milk. The milk was stirred and incubated at 35 °C for 40 min. Subsequently, the whey was separated from the clot, and its volume was measured. The cheese mass was pressed with a load weighing up to 5 kg for 16 h at a temperature of 8 °C. The amount of cheese produced (g) was then recorded to calculate the yield (%) for each production procedure. The moisture content of cheese was measured using a VIBRA MD-83 moisture analyzer (Shinko Denshi Co., Japan). Cheese yield (%) was calculated based on cheese weight (g) and milk volume (mL). The yield of solids (dry weight) of cheese (Y) was calculated using Eq. (2).

2 $$\:Y=M\times\:(1-\frac{H}{100\%})$$

Where M is the mass of cheese (g), and H is the moisture content of cheese (%).

### Statistical analyses and software

All measurements were performed in triplicate, and the results were defined as the mean of the three replicates. The mean values and standard deviations (SD) were calculated using GraphPad Prism V.8.0.1 software. Calculations of protein molecular weights and isoelectric points, analysis of chromatograms after sequencing, and primer design were performed using Vector NTI Advance 11 and SnapGene Viewer 8.0.1. Glycosylation sites were predicted using NetNGlyc 1.0 (http://www.cbs.dtu.dk/services/NetNGlyc/) and NetOGlyc 4.0 Server (http://www.cbs.dtu.dk/services/NetOGlyc/).

## Results

### Production and purification of Recombinant goat chymosin (rChCYM)

*ProcymCH* gene was integrated into the yeast shuttle vector, pPICZαA. After obtaining a transforming cassette and transforming *P. pastoris* GS115 cells, a total of ten colonies were obtained and subsequently subjected to PCR screening. The expression level was then checked in flasks and the strain-producing recombinant prochymosin was obtained. The prochymosin gene was integrated under the control of the methanol-inducible AOX1 promoter and the encoded prochymosin protein carried the N-terminal α-factor signal peptide from *Saccharomyces cerevisiae*, which was removed after secretory expression of the protein. After 72 h of cultivation of the transformed clones on YEP with 1.5% methanol, a clone with 52 units/mL of milk-clotting activity was selected. Recombinant goat prochymosin was isolated from 500 mL methanol-induced yeast culture for 5 days. By lowering the pH to 4.0 during cultivation, autocatalytic removal of the propeptide region occurred and prochymosin was activated to form active chymosin^2^. Recombinant goat chymosin was purified using ion exchange chromatography (Supplementary Figure S1).

The degree of purification, yield, and milk-clotting activity after each purification step of rChCYM is presented in Supplementary Table S1. According to the results, the highest loss of rChCYM was observed during cation exchange chromatography purification on SP-Sepharose FF, possibly due to increase in the sodium chloride concentration of the purified samples or a decrease in the binding capacity of the sorbent, possibly due to the presence of metabolites and other media components in the sample. In this case, the restoration of enzymatic activity following a reduction in salt concentration was not observed, despite the dilution of the solution. In the context of a reduction in sorbent binding capacity attributable to medium components and metabolites, the dialysis of the sample prior to purification is a viable option. However, the volume of culture liquid is substantial, which precludes the feasibility of this procedure. The utilization of sample concentration may offer a solution to this issue; however, it should be noted that there is a possibility of encountering similar losses during the process.

This effect has been observed by Rudometov et al.^21^; however, this purification method resulted in highly purified enzymes (Supplementary Figure S1), which is important for this research. The specific milk-clotting activity of rChCYM on cow and goat milk was found to be 7,680 ± 320 U/mg and 8,727 ± 390 U/mg, respectively. The proteolytic activity of rChCYM on hemoglobin was 7,769.2 ± 380 U/mg.

### Biochemical activity of rChCYM and rBtCYM

**Fig. 1:**
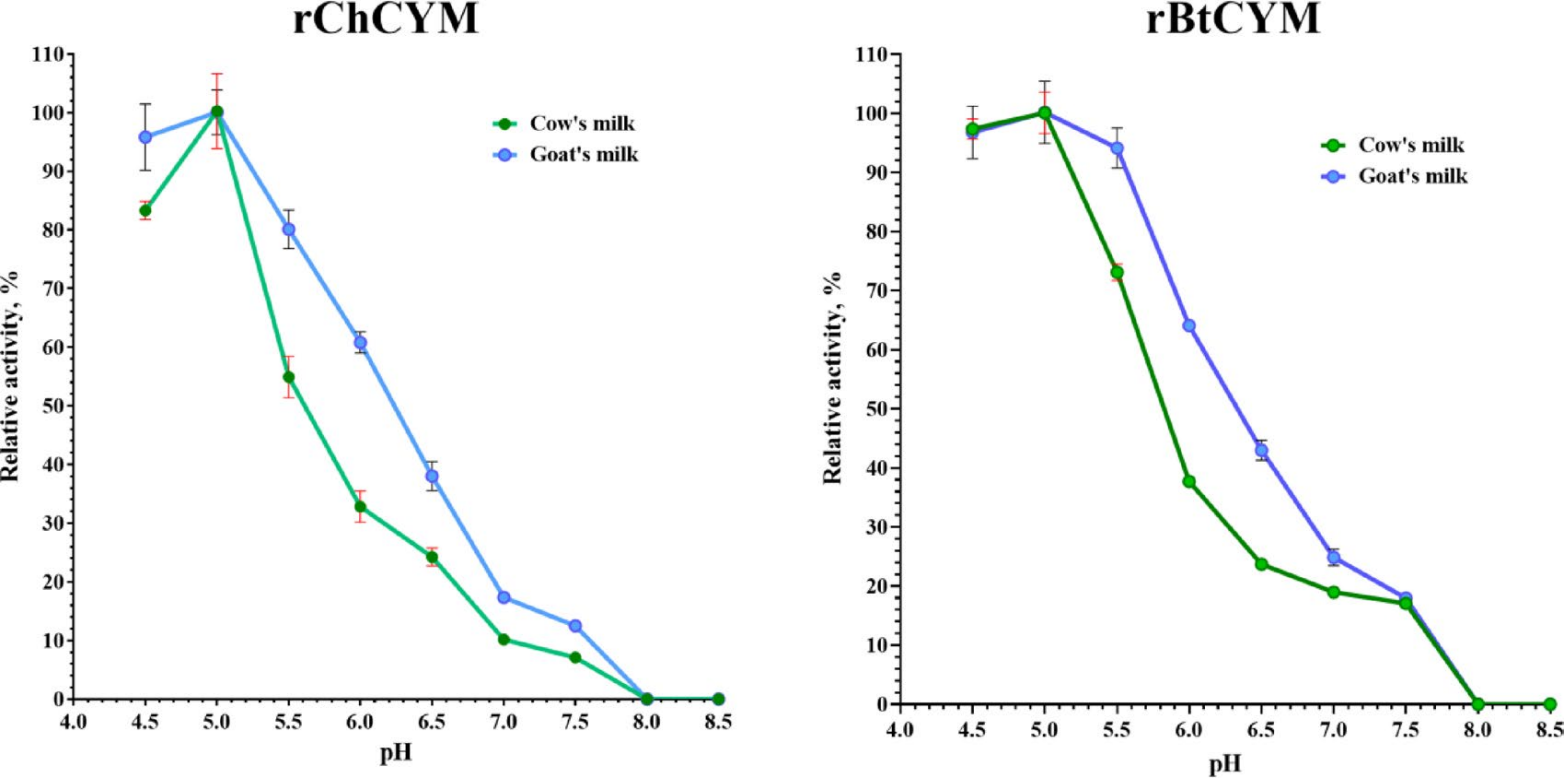
Substrate pH range for the milk-clotting activity of rChCYM and rBtCYM.

For comparative analysis, rBtCYM was used which was previously obtained under similar conditions^9^. A study on the dependence of the rChCYM and rBtCYM reaction rates on substrate pH demonstrated high milk-clotting activity (more than 80% of the maximum value) for both the chymosins in cow and goat milk at pH 4.5–5.0 (Fig. 1).

The milk-clotting activity of both the enzymes decreased with increase in pH. In the range of 6.5 to 7.5 the milk-clotting activity was not more than 40% of the maximum value, similar to the properties of native goat chymosin, as described by Kumar et al.^22^. Both enzymes were slightly more active (approximately 10%) in goat milk than those in cow milk at the same pH value in the range of 5.5–7.0. The milk-clotting activity of both enzymes was completely inhibited at pH ≥ 8.0.

**Fig. 2:**
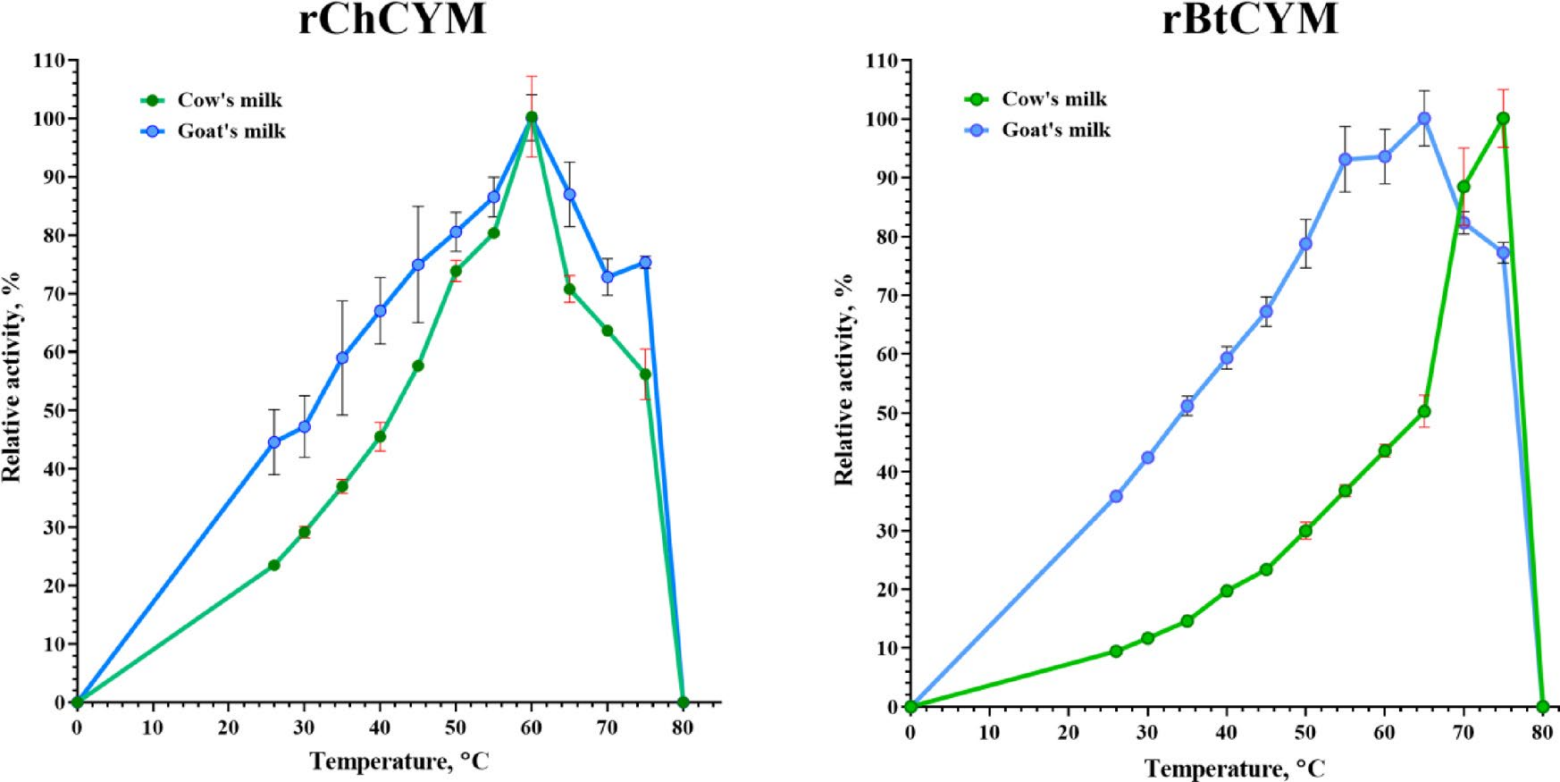
Temperature range for milk-clotting activity of rChCYM and rBtCYM.

rChCYM and rBtCYM showed different temperature-dependent activity profiles (Fig. 2). The highest activity of rChCYM was observed at 60 °C at pH 5.5 in both milk types, while rBtCYM exhibited the highest activity at 65 °C in goat milk while in cow milk was observed 50% activity, on the other hand 100% at 75 °C in cow milk with 77% in goat milk at pH 5.5. In the range of 45 °C and 65 °C, both enzymes retained 70% of their activity in goat milk and were completely inactivated at 80 °C in both milk types (Fig. 2).

**Fig. 3:**
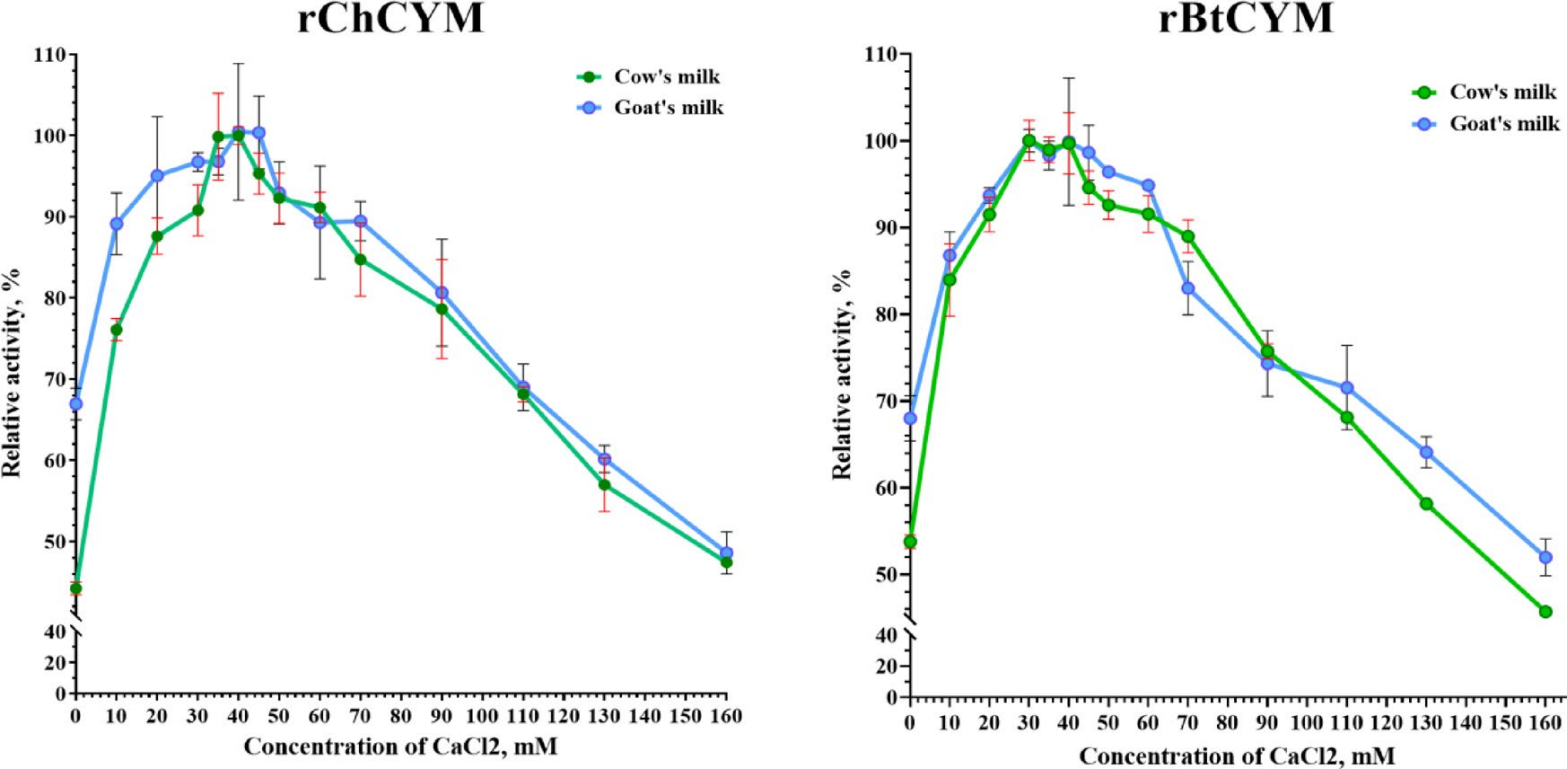
The impact of CaCl_2_ supplementation on the milk-clotting activities of rChCYM and rBtCYM.

An increase in the calcium concentration in goat or cow milk resulted in a decrease in coagulation time. rChCYM exhibited stable milk-clotting activity at calcium chloride concentrations of 10–90 mM, with maximum activity at 45 mM; rBtCYM exhibited stable activity in the concentration range of 10–70 mM, with maximum activity at 30 mM (Fig. 3).

The effects of Ca^2+^, Co^2+^, Ni^2+^, Fe^2+^, Ba^2+^, Zn^2+^, Cd^2+^, Mn^2+^, Mn^2+^, Li^+^, K^+^, and Na^+^ on the milk-clotting activities of rChCYM and rBtCYM are presented in Table 1.

**Table 1:** Effect of metal ions on the milk-clotting activities of rChCYM and rBtCYM (*n* = 3).

Metal ion	CaCl_2_ Concentration (mM)	Relative activity (%)			
		rChCYM		rBtCYM	
		Cow milk	Goat milk	Cow milk	Goat milk
Control	-	100 ± 4.6	100 ± 4.9	100 ± 4.4	100 ± 0.7
Ca^2+^	10	159 ± 6.9	131 ± 1.5	144 ± 0.6	137 ± 5.8
Co^2+^	10	73 ± 0.4	77 ± 6.3	64 ± 2.2	70 ± 2.0
Ni^2+^	10	0	14 ± 0.6	0	15 ± 0.3
Fe^2+^	10	130 ± 1.7	150 ± 5.5	127 ± 1.4	150 ± 11.8
Ba^2+^	10	171 ± 3.6	150 ± 5.8	149 ± 5.4	148 ± 6.2
Zn^2+^	10	42 ± 0.5	16 ± 0.6	61 ± 4.2	58 ± 4.0
Cd^2+^	10	49 ± 0.8	53 ± 2.8	150 ± 1.9	159 ± 7.3
Mg^2+^	10	119 ± 2.3	105 ± 4.3	105 ± 1.3	103 ± 4.6
Mn^2+^	10	177 ± 3.2	167 ± 5.1	153 ± 4.0	162 ± 6.8
Li^+^	10	75 ± 2.4	73 ± 5.7	70 ± 0.9	82 ± 0.8
K^+^	10	82 ± 2.3	82 ± 3.3	76 ± 1.2	95 ± 2.7
Na^+^	10	83 ± 1.7	89 ± 5.8	77 ± 1.1	91 ± 2.2

### Cheese production from goat and cow milk using rChCYM and rBtCYM

The possibility of using rChCYM for preparing experimental cheese from goat and cow milk was investigated. Table 2 shows the composition of fresh milk as determined using an analyzer. Calculations for the yields of laboratory cheese from goat and cow milk revealed that 522 and 540 g of cheese were obtained from 3 L of goat and cow milk, respectively. Table 3 presents the data extrapolated to 1 L of milk. The goat milk cheese had a slightly higher moisture content compared to the cow milk cheese, resulting in a softer and creamier texture. This difference in moisture levels could be due to the inherent properties of each type of milk used in the experiment. The higher moisture content in the goat milk cheese may have been due to its higher fat content compared to the cow milk cheese.

**Table 2:** Biochemical properties of goat and cow milk.

Milk	Fat (% w/v)	Proteins (% w/v)	Lactose (% w/v)	Ash (% w/v)	Solids (% w/v)	Density (g/cm^3^)
Goat	3.18	3.27	4.78	0.72	11.87	1.030
Cow	3.08	3.47	5.17	0.78	12.48	1.034

**Table 3:** A comparison of cheese production parameters using rChCYM in goat and cow milk.

Milk	Amount of Added Chymosin (U)	Whey Amount (L)	Postpress Cheese Yield (g)	Cheese Yield (%)	Moisture (%)	Yield of Solids (g)
Goat	1000	0.736	174	17.4	38.4	107.2
Cow	1000	0.73	180	18.0	32.0	122.4

### Pilot scale production of rChCYM in a bioreactor

**Fig. 4:**
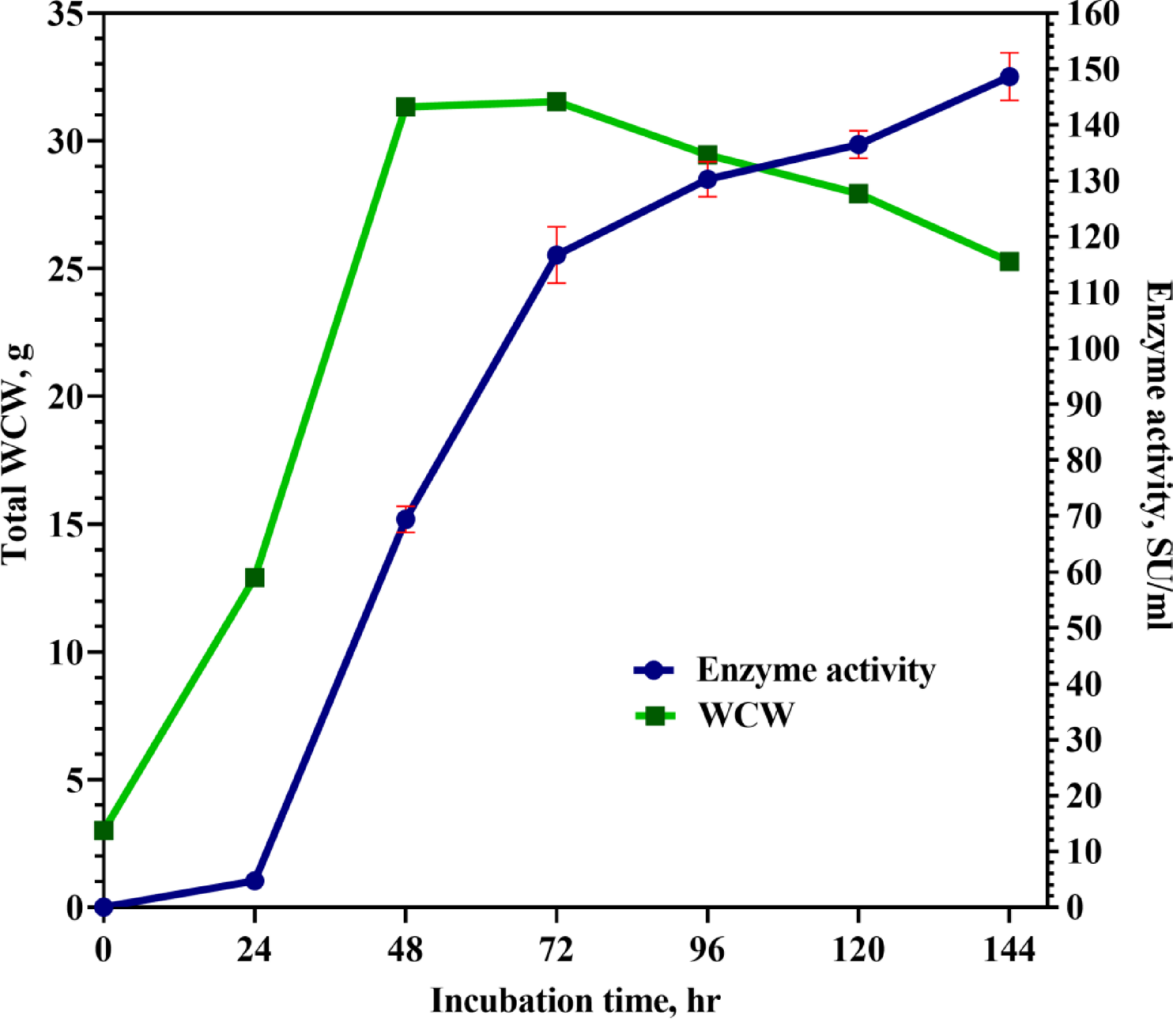
Pilot scale production of recombinant goat chymosin using yeast *Pichia pastoris*.

To investigate the possibility of producing rChCYM, a 10-L bioreactor was used to cultivate a yeast strain producer under submerged fermentation conditions. Figure 4 shows the dependence of wet cell weight gain and milk-clotting activity on cultivation time. AOX1, a tight regulator of gene expression in yeast, was found to be crucial for the production of rChCYM. No expression was detected before the induction phase and was initiated 24 h after induction. which led to the activation of AOX1 and subsequent expression of the desired protein. This regulation played a significant role in the successful cultivation of the yeast strain producer and the production of rChCYM in the bioreactor. A steady increase in growth was observed, peaking at approximately OD_600_ 24 after 72 h (Fig. 4). After 144 h of fermentation, the cell concentration reached 25 g/L, and the milk-clotting activity of the supernatant was 148 U/mL. The total yield of the recombinant enzyme was 5920 U per gram of cells which is comparable with the camel chymosin yield in *Pichia pastoris* cells which amounted to about 6280 U per gram^9^.

## Discussion

Rennet is the main enzyme responsible for milk coagulation during cheese making. Until the 1980 s, majority of the rennets were obtained from calf stomach. Subsequently, genetically engineered calf chymosin was produced by fermentation in a host microbial strain, which currently accounts for more than 90% of the rennet used, with advantages of being approved as a kosher and halal product^1^. Calf chymosin is effective in processing cow and goat milk, utilized in the production of soft brine cheeses. Goat milk cheeses markedly differ from cow milk cheeses because of variations in the casein ratio; goat milk, with its inherently lower αS1-casein content, possesses a higher proportion of β-, αS2-, and κ-casein compared to cow milk^23^. Consequently, goat cheeses tend to be less hard and less resistant to enzymatic cleavage than cow cheeses^24^.

In this study, goat chymosin was produced by fermentation of the recombinant yeast *P. pastoris* with an integrated *Capra hircus* chymosin gene. The goat preprochymosin gene was 1143 bp long, and its translation resulted in a 381-amino acid protein (Supplementary Fig. 2). A comparison of the amino acid sequences of calf (*Bos taurus*) and goat (*Capra hircus*) preprochymosins showed that the two proteins were 93% identical and differed in 23 amino acid residues: H15Q, P29S, R37H, V51I, E54K, R134H, T157I, V194I, D202N, R204H, G221E, D230N, L245V, K247Q, A261V, S310N, S313Y, Y331S, E337D, E338Q, G347S, H352Q, and Q353K. While the first five substitutions were located in regions not involved in the catalytic activity of chymosin (pre-and pro regions), the other remaining 18 substitutions could potentially influence the differences in milk-clotting and proteolytic activities of goat and calf chymosin.

Based on SDS-PAGE analysis, the molecular mass of rChCYM obtained from yeast was 42 ± 1 kDa, which differed from the theoretical mass of 38.6 kDa. The observed difference of 3 kDa can be attributed to glycosylation, due to the presence of mannose chains in recombinant goat prochymosin. *P. pastoris* cells possess a well-developed N-glycosylation system and attach an average of 8–14 mannose residues per side chain of the polypeptide^25^. Analysis of the amino acid sequence of *Capra hircus* prochymosin showed that the protein possesses one possible N-glycosylation site, Asp333-His334-Ser335, through which glycosylation occurs, analogous to the glycosylation of calf and camel prochymosins obtained in *P. pastoris*^8,9^. The secondary structure of aspartic acid proteases, to which chymosin belongs is predominantly a β-chain with several short α-helices^26^. The specificity of chymosin to κ-casein is due to the conformational state, wherein two Asp residues form the active center in the chymosin structure (D92 and D274 as numbered in Supplementary Fig. 2) and are embedded in the active site of the enzyme. No differences in the amino acid sequences of the active sites of both enzymes were observed; therefore, the catalytic properties of rChCYM and rBtCYM were similar.

Biochemical analyses revealed that rBtCYM and rChCYM exhibit similar parameters owing to the high degree of enzyme homology. Both enzymes exhibit maximum activity under acidic conditions (pH 4.5–5.5), where the activities did not reduce below 40%; however, with increasing pH the milk-clotting activity decreased sharply. A sharp decrease in enzyme activity at pH ≥ 5.8 has been reported previously^27^. This is because chymosins are acidic proteases whose action is restricted to the stomach of animals, where the acidity of the gastric juice maximizes their proteolytic and coagulation abilities.

Both enzymes showed a strict dependence on the calcium concentration. This can be explained by the fact that during coagulation, calcium ions form bridges between the para-κ-casein micelles; thus, precipitation of the micelles is accelerated^22^. Chymosin activity decreased at both low (< 10 mM) and high concentrations (80 mM >) of calcium chloride in milk. In the absence of calcium chloride, goat chymosin showed a relative milk-clotting activity of less than 45% and 67% in cow and goat milk, respectively. This outcome is important when using pasteurized milk with low calcium content^28^. Other metal ions also influenced the milk-clotting activities of rBtCYM and rChCYM. Cd^2+^ ions decreased rChCYM activity by 50%, but promoted rBtCYM activity by 50%. The addition of Fe^2+^, Ba^2+^, and Mn^2+^ increased milk-clotting activity by 1.3–1.7-fold, while Co^2+^, Li^+^, K^+^, and Na^+^ ions reduced the activities to 60–70% of the control value. The presence of Zn^2+^ and Ni^2+^ ions significantly inhibited enzyme activity. Nickel ions are known to inhibit the activity of elk^8^ and camel^10^ chymosins, which may be a characteristic of all chymosins.

Substrate temperature had a significant effect on milk-clotting activity. The enzyme activities increased gradually with temperature, and differences in the temperature sensitivities of rBtCYM and rChCYM reactions were observed. Here, rChCYM showed maximum activity at 60 °C on both goat and cow milk at pH 5.5, while rBtCYM maximum activity depended on the milk type, with 65 °C and 70 °C in goat and cow milk at pH 5.5, respectively. A comparison of milk-clotting activities showed that rChCYM activity was 38% higher than that for rBtCYM in goat milk^9^.

Conceivably, chymosin hydrolyzes its own casein better than that from other species. Goat chymosin differs from both calf and camel chymosins, which exhibit low activity in goat milk compared to those in cow and sheep milk^9^. Goat milk contains lesser casein (particularly less αS1-casein) and larger casein micelles than cow’s milk; these characteristics are presumably responsible for the poor consistency of goat milk clots during acid or enzymatic coagulation^23^. In contrast, relatively higher amounts of caseins and minerals are potentially responsible for the stronger acid or rennet clotting in sheep milk compared to that in goat or cow milk^23^ (Table 2).

Our studies showed that rBtCYM activity was 40, 14, and 55% higher in cow, sheep, and camel milk, respectively, than rChCYM activity (Table 4). Cow milk clot formation and density were similar when either goat or calf chymosin was used. In contrast, when goat milk was used, goat chymosin exhibited better milk-clotting time and faster clot thickening than those using calf chymosin, similar to the findings reported by Libouga et al.^29^. The milk-clotting activities of both the enzymes in mare milk were identical. Cheese quality depends largely on the rate, extent, and nature of proteolysis and lipolysis, which are the two main biochemical processes involved in cheese aging^24^. Excessive proteolysis leads to excessive melting of the cheese with the formation of an excessive amount of free oil, as a result of which the cheese becomes unsuitable for mechanical processing (cubbing, slicing, or shredding); excessive proteolysis also reduces the shelf-life of cheese^1^.

**Table 4:** The milk-clotting activities of purified rChCYM per 1 mg of enzyme were tested on reconstituted cow, goat, ewe, camel, and mare milk in comparison to rBtCYM.

Chymosin	Milk type				
	Cow	Goat	Ewe	Camel	Mare
rChCYM (U/mg)	7680 ± 320	8727 ± 390	12,800 ± 590	4467 ± 180	126 ± 3.3
rBtCYM^a^ (U/mg)	12,854 ± 610	5385 ± 250	14,811 ± 720	10,013 ± 430	130.6 ± 5.5

^a^ – the results were used from^9^.

Comparative proteolytic activities showed that rChCYM exhibits 28% lower proteolytic activity than that of rBtCYM^10^. The ratio of milk clotting (C) activity to total proteolytic (P) activity (C/P) for of rChCYM and rBtCYM were 0.9 and 1.2, respectively. Thus, both enzymes are specific proteases with milk-clotting activity.

During goat chymosin production in a lab-scale bioreactor, the protein was expressed only after 24 h of induction, indicating strict regulation of goat chymosin expression under the control of the AOX1 promoter. Analysis of the enzymatic activity of chymosin produced in the early stages of induction showed that production is completely dependent on induction, and the enzyme produced even in the early stages is conformationally correct and functionally active. Analysis of the fermentation results showed that 72–96 h was the optimal time for culture collection in the bioreactor. In addition, in the bioreactor study, the yield of the recombinant enzyme increased by 64%. These results are consistent with those of other studies^27,30^, in which a similar increase in cell density and enzyme activity has been noted.

Goat milk-based cheese making is a complex process owing to several factors, including the quality of the processed milk, mainly fat and casein; the recovery of these nutrients in the curd; water retention in cheese; and the overall efficiency of cheesemaking, all of which affect the percentage yield of cheese. In cheese-making experiments, the action of goat chymosin differed depending on the milk type. The cheese yields of both types were similar but differed in dry matter content by 12% (Table 3) due to the higher moisture content of goat cheese. Goat cheese differs from cow cheese in terms of moisture content, texture, flavor, and ripening. Owing to differences in the size of fat globules (smaller fat droplet size) and protein composition, the cheese in goat milk has a softer texture than that in cow milk.

In summary, the activity of yeast-derived rChCYM on goat milk demonstrates its potential as a milk-clotting enzyme in goat milk cheese-making technologies. This statement is supported by the higher specific activity of rChCYM (8727.0 ± 0.39 U/mg) than those of recombinant calf (5385 ± 0.25 U/mg) and camel chymosin (7850 ± 0.34 U/mg) on goat milk^9^, with low proteolytic activity (7769.2 ± 0.38 U/mg) relative to recombinant calf chymosin^9^. Since goat milk cheese is primarily produced by artisan cheesemakers, it is important to have a wider range of enzymes that do not significantly impact clot quality. For high-quality goat milk cheese, this helps maintain similar cheese yields. Experiments on cheese production from goat milk confirmed the potential of rChCYM as a milk-clotting enzyme for processing goat milk. The yeast expression system is a valuable tool for enabling efficient fermentation in producer strains, allowing cost-effective biotechnological production of recombinant chymosin (rChCYM). However, scaling up rChCYM production for industrial use may involve challenges such as optimizing yield, improving purification efficiency, and navigating regulatory requirements. On the other hand, exploring potential modifications to rChCYM’s structure could enhance its milk-clotting activity and proteolytic properties, tailoring it for improved performance in cheese-making processes. Addressing these factors would strengthen its practical application in the dairy industry.

In this study, recombinant goat chymosin was obtained from *Pichia pastoris* yeast and was first biochemically described in goat milk and compared to recombinant calf chymosin. The milk-clotting activity of purified rChCYM was maximum in goat milk compare to those in cow, sheep, camel and mare milk. In addition, goat and calf recombinant chymosin, demonstrated the highest activity in the pH range of 4.5–5.0. The ideal temperature for enzyme activity of goat chymosin was 60 °C for both milk types, whereas calf chymosin shows maximum activity at 65 °C and 75 °C for goat and cow milk, respectively. The milk-clotting activity of both enzymes was inhibited by nickel ions and depended strongly on the calcium chloride concentration. The fermentation yield of rChCYM-producing strain was 148 U/mL. Cheese production experiments further revealed that rChCYM is superior to rBtCYM in coagulation properties and is the preferred milk-clotting enzyme in goat milk processing technologies.

## Electronic supplementary material

Below is the link to the electronic supplementary material.

Supplementary Material 1

## Data Availability

All data generated or analysed during this study are included in this published article (and its Supplementary Information file).
