# Supplementary material for: Comparative biochemical properties of recombinant goat and calf chymosins and their implications in dairy processing
Source: Sci Rep. 2025 Jul 21;15:26502. doi: 10.1038/s41598-025-11833-x (PMC12280187; doi:10.1038/s41598-025-11833-x)
Supplement: Supplementary file 1 — Supplementary Material 1 [file 41598_2025_11833_MOESM1_ESM.pdf]

Comparative Biochemical Properties of Recombinant Goat and Calf Chymosins: Implications in Dairy Processing

Zhiger Akishev[1,3], Madina Auyez[1], Annelya Tursunbekova[1,2], Bekbolat Khassenov[1,3*]

[1] *National Center for Biotechnology, 13/5 Korgalzhyn Road, Astana, 010000;Kazakhstan*
[2] *S.Seifullin Kazakh Agro Technical Research University, 62 Zhenis Avenue, Astana, 010001, Kazakhstan;*
[3] *"GenLab" LLP, 19/1, 69, M. Gabdullin Street, Astana, 010000, Kazakhstan.*
***** Correspondence: khassenov@biocenter.kz

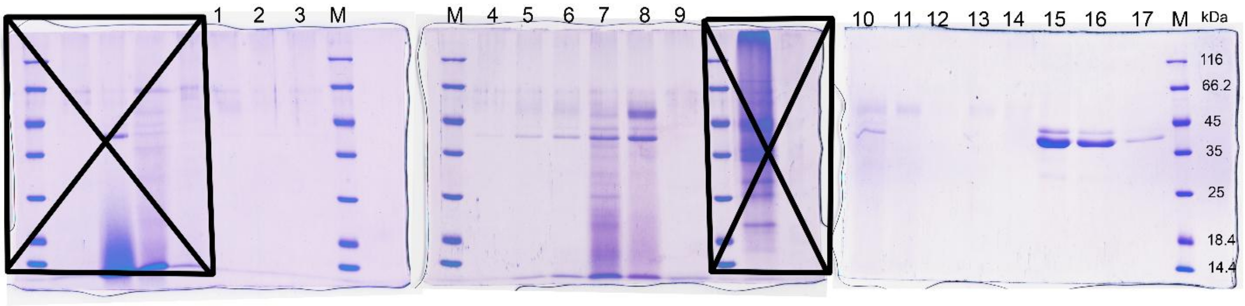

Supplementary Figure 1. Sodium dodecyl sulphate-polyacrylamide gel electrophoresis analysis of rChCYM purification. Lane 1, Uninduced cultural medium; Lane 2, Induced cultural medium after 120 h; Lane 3 SP-Sepharose unbound fraction; Lanes 4–9, SP-Sepharose-purified fractions; Lane 10, Combined and diluted fraction before loading on Q Sepharose; Lane 11, Unbound flow through Q Sepharose; Lanes 12–17, Q-Sepharose-purified fractions; M, molecular-weight markers (#26610, Thermo Scientific).

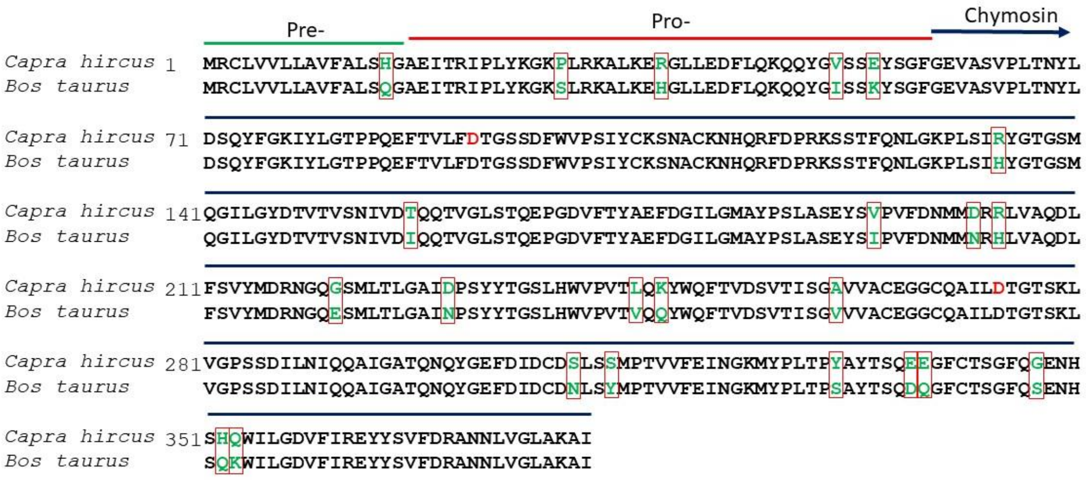

Supplementary Figure 2. Amino acid sequences of calf and goat prochymosins. Amino acid substitutions of the two proteins are highlighted in green, and Asp residues in the active center are highlighted in red.

Supplementary Table 1. Purification of rChCYM

**Purification step**	**Milk-clotting activity* (U)**	**Total protein (mg)**	**Specific activity (U/mg)**	**Relative purification fold**	**Recovery (%)**
Cultural media	45,048	73.5	613	1	100
Cation exchange chromatography	32,388	11.55	2804	4.6	71.9
Anion exchange chromatography	24,024	3.128	7680	12.5	53.3

* Milk-clotting activity was measured in reconstituted skim cow milk.
